# Efficacy and safety of Mavacamten for symptomatic Hypertrophic cardiomyopathy – an updated Meta-Analysis of randomized controlled trials

**DOI:** 10.1016/j.ijcha.2024.101467

**Published:** 2024-07-13

**Authors:** Irfan Ullah, Syeda Tayyaba Rehan, Zayeema Khan, Syed Hasan Shuja, Muhammad Hamza Shuja, Muhammad Irfan, Karthik Gonuguntla, M Chadi Alraies, Pratik Aggarwal, Sameer Raina, Yasar Sattar, Muhammad Sohaib Asghar

**Affiliations:** a Department of Internal Medicine, Kabir Medical College, Gandhara University, Peshawar, Pakistan; b Department of Internal Medicine, Khyber Teaching Hospital, Peshawar, Pakistan; c Department of Internal Medicine, Dow University of Health Sciences, 74200, Karachi, Pakistan; d Department of Internal Medicine, Wellstar Spalding Medical Center, Georgia, USA; e Department of Cardiovascular Medicine, West Virginia University, WV, Morgantown, USA; f Department of Cardiovascular Medicine, Detroit Medical Center, Detroit, MI, USA; g Department of Cardiovascular Medicine, Stanford University, CA, USA; h Department of Internal Medicine, AdventHealth Sebring, FL, USA; i Division of Nephrology and Hypertension, Mayo Clinic, MN, USA

**Keywords:** Mavacamten, Hypertrophic Cardiomyopathy, NYHA, Safety, Efficacy

## Abstract

Hypertrophic cardiomyopathy (HCM) is an autosomal dominant disorder with risk of sudden cardiac death (SCD) in children and adolescents. Mavacamten, also referred to as MYK-461, a myosin inhibitor of cardiac myocytes is studied in symptomatic HCM. The safety and efficacy of this medication is not well studied in pooled *meta*-analysis. Online database search was performed from inception to September 2023. We selected randomized clinical trials that compared Mavacamten with placebo/guideline medical treatment for HCM. We studied safety outcomes (Serious adverse events (SAEs), treatment emergent adverse events (TEAs) and Atrial fibrillation). Functional status of patients was assessed as New York Heart Association (NYHA) Classification improvement of at least + 1 grade, Kansas City Cardiomyopathy Questionnaire Clinical Summary Score (KCCQ-CSS) change from baseline). Relative risk ratios were used in randomized model using *Review Manager Version 5.4 statistical software.* A total of 4 RCTs comprising 503 patients were included in *meta*-analysis. On random effect model, we found that HCM patients that received Mavacamten had significant symptomatic improvement as depicted by improvement in NYHA class by at least + 1 grade (RR = 2.15; P < 0.0001) and KCCQ CSS score improvement (MD = 8.38; P < 0.00001) as compared to placebo arm. There was no statistically significant difference in SAEs (RR = 0.87; P = 0.69) and atrial fibrillation onset (RR = 0.80; P = 0.73) between HCM and placebo arm. The studies had low heterogeneity/publication bias. Mavacamten can improve symptoms in HCM patients, and can be additive to other alternative regimen in HCM patients with no statistical significance of risk of SAE or atrial fibrillation onset as compared to placebo.

## 1 Introduction

Hypertrophic cardiomyopathy (HCM) is an autosomal dominant disorder with variable presentation given incomplete penetrance and variety of mutations in sarcomere genes [1]. HCM is the most common hereditary cardiovascular condition and is a major cause of sudden cardiac death (SCD) in children and adolescents [2]. Asymmetric Left ventricular hypertrophy (LVH) is the hallmark of HCM, and is prevalent at the basal interventricular septum [3]. HCM also exhibits mitral, sub-valvular regions, intra-cavitary and outflow tract manifestations. There is myocardial disarray, microvascular pathology, decreased compliance, and cardiac fibrosis that can lead to these manifestations [4]. A hallmark of HCM can be presence of left ventricular outflow tract (LVOT) obstruction that can lead to symptomatic presentation with presentation of congestive heart failure (CHF), atrial fibrillation (AF), stroke, and an increased cardiovascular mortality [5]. The primary focus of treatment is symptomatic improvement and relief of obstructive HCM (HOCM) [6].

The management of symptomatic HOCM included medical management with negative inotropy/chronotropy by use of beta-blockers (BB) and calcium channel blockers (CCB) [7]. BB are typically the first-line therapy for symptomatic HOCM, while CCB’s are second line adjunctive regimen [8]. However, these drugs provide symptomatic relief to patients but do not address the pathophysiological principles of HCM, necessitating the development of innovative therapies. For patients who are refractory to medical therapy and have significant LVOT obstruction, procedures such as surgical myomectomy, or transcatheter alcohol septal ablation are necessary [9]. However, the invasiveness of these procedures contributes to increased morbidity and mortality and the requirement for highly specialized medical centers to conduct such procedures is not universally available [10]. This highlights the crucial need for a long-term treatment solution that can target the root causes of the condition.

Mavacamten, also referred to as MYK-461, a novel cardiac myosin inhibitor approved by US Food and Drug Administration (FDA) in 2022 for use in symptomatic Obstructive Hypertrophic Cardiomyopathy (HOCM) After EXPLORER HCM trial [11], [12]. HCM is often driven by specific myosin missense mutations that disrupt the normal intramolecular interactions that promote the folded state of myosin. This disruption leads to an excess of available myosin heads, resulting in a hypercontractile state. In contrast, Mavacamten, a reversible, selective allosteric inhibitor of cardiac myosin ATPase, operates by shifting the equilibrium toward the off-folded state. As a result, it brings about a dose-dependent reduction in contractility. a mechanism involved in HCM pathogenesis [13].

To date, only 4 randomized controlled trials (RCTs) have evaluated the efficacy and safety of Mavacamten in patients with HCM compared to placebo [6], [11], [14], [15]. Hence we conducted a *meta*-analysis, utilizing randomized controlled trials (RCTs), to evaluate both the effectiveness and safety of Mavacamten in comparison to a placebo for the targeted medical management of hypertrophic cardiomyopathy.

This *meta*-analysis adhered to the Preferred Reporting Items for Systematic Review and Meta-Analysis (PRISMA) guidelines [16] and was performed in accordance with the structure designed by the Cochrane collaboration [17]. The research has been registered on *PROSPERO [ID CRD42023464476].* An extensive literature search encompassing MEDLINE, Cochrane Central, ScienceDirect, *ClinicalTrials.gov*, and Google Scholar databases has been done from their inception up to September 2023, without imposing any limitations related to time, language, or sample size. The complete search strategy description used in each of the databases is given in Supplementary Table S1. The bibliographies of pertinent review articles, internet databases like clinicaltrials.gov, and preprint sites like *MedRvix* were also screened for gray literature.

The systematic search yielded articles, which were then imported into *EndNote Reference Manager (Version X7.5; Clarivate Analytics, Philadelphia, Pennsylvania).* Within *EndNote*, we systematically screened for and removed any duplicate entries.

Two reviewers (SHS and STR) independently evaluated the remaining articles first by reviewing their titles and abstracts, after which they conducted a comprehensive review of the full texts to confirm their relevance. Any disagreements were resolved through group discussion. Studies were selected if they met the following predefined eligibility criteria a.) randomized controlled trials (RCTs) with the intervention group receiving Mavacamten and the control group receiving the placebo; (b) adult patients (≥18 years) with HOCM or non-obstructive HCM (HNCM). Duplicate records, case reports, commentaries, and editorials were excluded. Animal studies were also excluded from this paper.

Data on baseline characteristics, study type, study year, sample size, age, and gender from the eligible articles were extracted onto a standard Excel sheet. The primary outcomes of interest were symptomatic improved reported as New York Heart Association (NYHA) class improvement of at least grade 1 or more and Kansas City Cardiomyopathy Questionnaires-clinical summary score (KCCQ CSS) change from baseline. The secondary outcomes were: any treatment emergent adverse events (TAEs) including palpitations, dizziness, nausea, dyspnea, and fatigue during treatment or at follow-up (16 or 30 weeks), any serious adverse events (SAEs) including syncope, stress cardiomyopathy, atrial flutter, atrial fibrillation, sinus node dysfunction, systolic dysfunction, arthritis, mental status changes, and renal failure during treatment or at follow-up (24 or 30 weeks), and onset of paroxysmal atrial fibrillation (PAF) during treatment or at follow up (16 or 30 weeks). Two separate reviewers (MHS and SHS) conducted an evaluation of the methodological quality of the included RCTs employing the Cochrane risk of bias assessment tool [18].

We utilized the original data to compute risk ratios (RRs) and their corresponding 95 % confidence intervals (CIs) using a random effects model for the dichotomous outcomes. For continuous outcomes, we conducted a *meta*-analysis of the weighted mean difference (WMD) and its accompanying 95 % confidence intervals (CIs), also employing a random effects model. Heterogeneity among the studies included in the analysis was evaluated using Higgins I^2^ statistics. We classified I^2^ values as follows: mild heterogeneity for I^2^ between 25 % and 50 %, moderate heterogeneity for I^2^ between 50 % and 75 %, and severe heterogeneity for I^2^ exceeding 75 % [19]. Outcomes from studies that reported a high percentage of heterogeneity were subjected to sensitivity analysis to explore the effect of each study on the pooled estimate. Subgroup analysis was performed for two outcomes: KCCQ CSS change from baseline and NYHA class improvement on the basis of follow up duration (16 and 30 weeks). As the number of studies included for all outcomes was fewer than ten, we did not perform tests to assess funnel plot asymmetry in accordance with the Cochrane Guidelines [17]. In all cases, a p-value of < 0.05 was considered statistically significant. The statistical analysis was performed using *Review Manager Version 5.4 Cochrane Collaboration.*

**Fig. 1:**
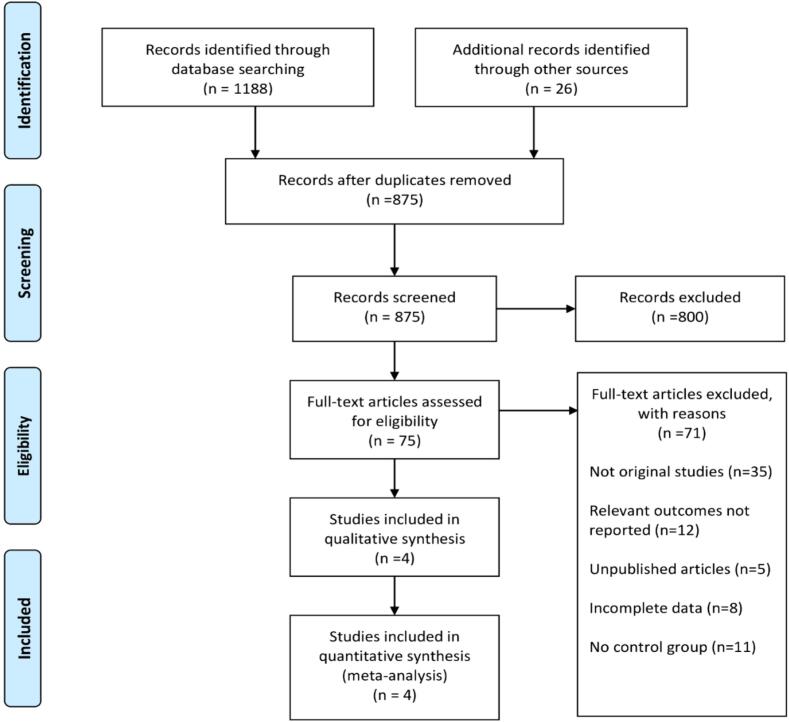
PRISMA flow chart showing study selection.

We conducted an online database systematic search including 5 electronic databases that showed 1,214 studies, out of which 875 records were left after removing the duplicate studies. 800 studies were further eliminated after the title and abstract screening. After a full-text review of 75 articles which were assessed for their eligibility, 71 articles were excluded which did not meet the inclusion criteria. Consequently, 4 RCTs were finalized for this *meta*-analysis [6], [11], [14], [15]. A complete literature search has been highlighted in the *PRISMA flowchart* **(**Fig. 1**).**

A total of 503 patients were enrolled in these 4 RCTs, out of which 273 patients belonged to the Mavacamten supplemented group, and 230 patients belonged to the control group. The mean age of the patients ranged from 51.9-60.35 years with an average of 56.1 years. The mean percentage of males was 57.4 % of the total population. Study characteristics and patients' baseline characteristics have been mentioned in detail in Table 1.

**Table 1:** Baseline demography and characteristics of included studies.

Author (year)	RCT Name	Trial Population (n)	Intervention (n)	Control (n)	Age Mean (S.D) & Gender (M/F)	Baseline NYHA class (n)		Baseline KCCQ CSS Score; Mean (SD)		Follow-up Duration (weeks)
						Intervention	Placebo	Intervention	Placebo	
CAROLYN (2020)	MAVERICK-HCM	59 Non obstructive HCM	19 with mavacamten 200 ng/dl 21 with mavacamten 500 ng/dl	19; Placebo	53.8 (16.0) & (25/34)	33 class II7 class III	13 class II6 class III	NA		16 weeks for echo outcomes,24 weeks for TAEs, SAEs, and Afib
DESAI (2022)	VALOR-HCM	112 obstructive HCM patients	56; Mavacamten 2.5, 5, 10 or 15 mg	56; Placebo	60.35 (12.5) & (57/55)	4 class II, 53 class III or higher	4 class II52 class III or higher	69.5(16.3)	45.9(19.9)	16 week
OLIVOTTO (2020)	EXPLORER-HCM	251 obstructive HCM patients	123; Mavacamten 2.5, 5, 10, or 15 mg	128; Placebo	58.5(11.1) & (149/103)	88 class II,35 class III	95 class II33 class III	NA		30 week
ZHUANG TIAN (2021)	EXPLORER-CN	81 obstructive HCM patients	54; Mavacamten 1,2.5,5,10 or 15 mg	27; Placebo	51.9 (12.0) & (58/23)	44 class II, 10 class III/	18 class II9 class III /	82.4(16.9)		16 week

Abbreviations: RCT: Randomized Controlled Trial; HCM: Hypertrophic Cardiomyopathy; NYHA:New York Heart Association; KCCQ-CSS: Kansas City Cardiomyopathy Questionnaire.

**Fig. 2:**
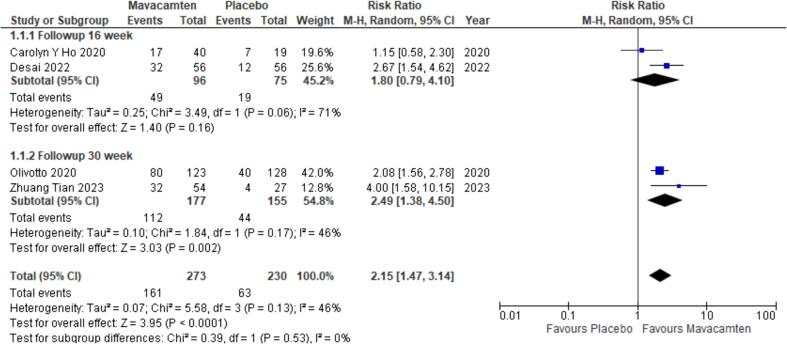
Subgroup analysis depicting improvement in NYHA class of at least 1 or more in patients with HCM from baseline to follow-up.

The four included RCTs reported the safety and efficacy of Mavacamten vs placebo on patients with HOCM and HNCM. All four RCTs provided data for the effects of Mavacamten on the rate of NYHA improvement in HCM patients. A significant improvement in NYHA score was observed in the Mavacamten patient group as compared to the control group (RR = 2.15; P < 0.0001) with mild heterogeneity present amongst the four studies (I^2^ = 46 %). Subgroup analysis was conducted on the basis of follow-up time. Two studies followed up with the patients after 16 weeks [14], [15] and two studies had a follow-up time of 30 weeks [6], [11]. The results only indicated a significant improvement in NYHA score in the 30 week follow up subgroup (RR = 2.49; P = 0.002) (Fig. 2).

**Fig. 3:**
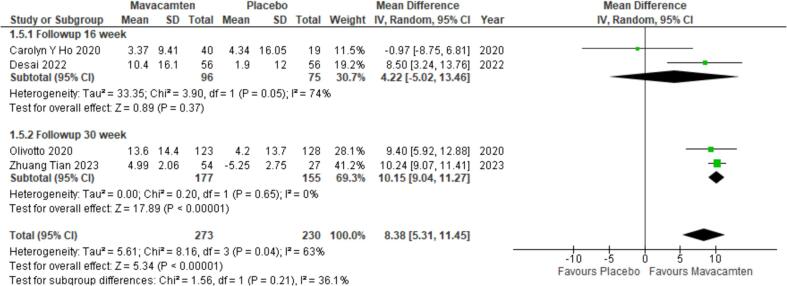
Subgroup analysis forest plot showing mean difference of KCCQ CSS score in patients with HCM from baseline to follow up.

All included studies compared data on the change in KCCQ CSS score from baseline in HCM patients [6], [11], [14], [15]. A significant improvement in the mean difference of KCCQ CSS score was observed in the mavacamten patient group as compared to the control group (MD = 8.38; P < 0.00001) with high heterogeneity present amongst the four studies (I^2^ = 63 %). Subgroup analysis was conducted on the basis of follow-up time. Two studies followed up with the patients after 16 weeks [14], [15] and two studies had a follow up time of 30 weeks [6], [11]. The results only indicated a significant improvement in the mean difference of KCCQ CSS score in the 30-week follow-up subgroup (MD = 10.15; P < 0.00001) (Fig. 3). Sensitivity analysis was conducted to reduce heterogeneity by removing one study [15] that greatly reduced heterogeneity and significantly improved the mean difference of KCCQ CSS score (MD = 10.08; P < 0.00001) (Supplementary Figure 1) [15]. This heterogeneity can be attributed to the differing population of the trial consisting of non-obstructive HCM pat.

**Fig. 4:**
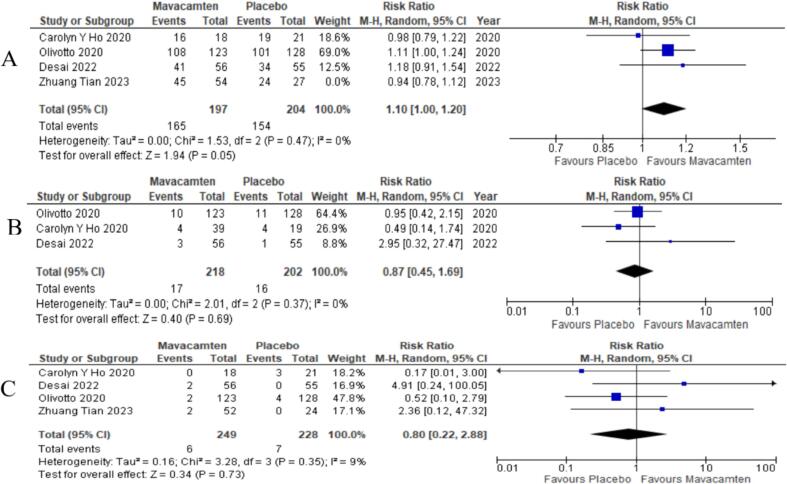
Forest plots depicting (A) patients with any treatment emergent adverse events (TAEs); (B) patients with serious adverse events (SAEs) during treatment or at follow-up of 24–30 weeks, (C) Patients with Atrial Fibrillation during treatment or at follow-up of 16–30 weeks.

All four studies published data on the treatment of emergent adverse events (TAEs) in HCM patients. The results were found to be insignificant in the mavacamten treatment group as compared to the control group (Supplementary Figure 2). (RR = 1.05; P = 0.32) [6], [11], [14], [15]. Sensitivity analysis was conducted to reduce the heterogeneity (I^2^ = 24 %) which yielded a significant difference in the interpretation of results (RR = 1.10; P = 0.05) (Fig. 4A).3 out of four RCTs provided adequate data regarding SAEs after a follow up of 24–30 weeks [11], [14], [15]. An insignificant difference was observed between both the groups (RR = 0.87; P = 0.69) (Fig. 4B). Four RCTs provided data regarding the occurrence of PAF during treatment or at a follow-up of 16–30 weeks [6], [11], [14], [15]. No significant differences were observed between the mavacamten and the control group (RR = 0.80; P = 0.73) (Fig. 4C).

**Fig. 5:**
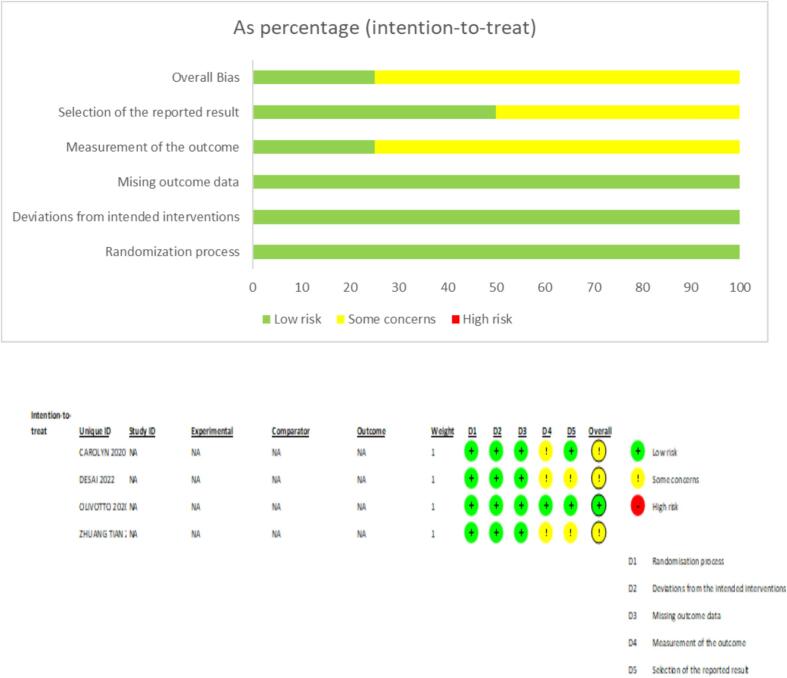
A-B: Risk of Bias Summary and Graph.

The majority of the included studies reported an overall low risk of bias, enhancing the authenticity of this *meta*-analysis. Out of the 4 included studies, 3 studies reported outcome measurement bias which was the only main factor that hindered the quality of included studies [6], [14], [15]. Additionally, the trials conducted by Desai et al. and Zhuang Tian et al. reported selection of reported results bias [6], [14]. On assessment, the overall quality of all the included studies was declared high. The detailed results of the quality assessment are listed in Fig. 5A-B.

Our analysis of 503 HCM patients provides compelling evidence for the efficacy and safety of mavacamten. This *meta*-analysis highlighted a significant improvement in NHYA and KCCQ CSS clinical outcomes in both obstructive and non-obstructive HCM patients after mavacamten use. The safety profile of the mavacamten intervention when compared to the placebo group was found to be satisfactory. The difference between the occurrence of TAEs in the intervention and the control group was found to be minimally significant (P = 0.05). Events of SAEs and atrial fibrillation were similar among mavacamten and placebo (P > 0.05).

In comparison to earlier reviews that have been published on the subject, this analysis is significantly distinct. The validity of the results from a Rad et al. *meta*-analysis from 2023 is further weakened by the study's use of only two studies for each outcome [20]. The improvement of cardiovascular NYHA grade of one or more, which has been examined by other research and also supports the findings of the current study [21], [22], is one of the study's main objectives. Furthermore, Affas Z et al.'s *meta*-analysis from 2022 combined data from ongoing studies, rendering the review's findings invalid because the trials' follow-up times haven't yet reached their conclusion. The present review pools data from 4 completed RCTs which provides substantial evidence supporting the efficacy of mavacamten [21]. Recently the use of mavacamten for the treatment of HOCM and HNCM has gained traction after the completion of VALOR-HCM, EXPLORER-HCM, MAVERICK-HCM, and the EXPLORER-CN trials [6], [11], [14], [15]. The latter trial conducted in Chinese patients hasn’t yet been pooled in any review or analysis which has diversified the findings of the present study [6]. Mavacamten reduces contractility and increases ventricular compliance by directly decreasing sarcomere force output via reversibly blocking beta-cardiac myosin binding to actin [23].

Mavacamten’s ability to improve cardiac function and exercise ability in HCM patients can be attributed to improving cardiac structure, reduction in brain natriuretic peptide levels (BNP), and hs-CTN1 [24]. A noteworthy addition in establishing mavacamten’s efficacy in improving the LVOT gradient is its role in reducing the need for septal reduction therapy (SRT) in symptomatic HCM patient population which has been proved by the key findings of the EXPLORER-HCM study [11]. This has further been replicated by the VALOR-HCM trial which associated improvements in cardiac function and associated biomarkers to a decreased need for surgical intervention and have also been efficacious for HOCM patients who are otherwise candidates for septal reduction therapy [14], [25].

The inclusion and exclusion criteria used in the four included RCTs represent a significant outlier among the included studies in the current investigation that must be taken into account. The patient population in the EXPLORER-HCM and VALOR-HCM studies is already receiving a background therapy of beta-blockers and calcium channel blockers, which may have prevented mavacamten from having an impact on cardiac myosin channels [25]. In order to further validate the trial's findings, individuals receiving either beta blocker or diltiazem therapy were excluded from the recently published RCT by Zhuang Tian et al. [6].

It's important to take into account Mavacamten's safety profile even though it seems to have a lot to offer in the treatment of HCM. Evidence supporting post or during intervention adverse effects of mavacamten is limited. Our analysis showed insignificant results for SAEs and atrial fibrillation although post-sensitivity analysis for TAEs showed minor significant differences between the mavacamten and placebo groups. These findings are corroborated by the individual trials which have been pooled in the analysis. Although our study did not signify any major adverse effects of mavacamten, long term extension trials with longer follow-up periods are currently underway. Compared to the relatively short follow-up durations of current trials like MAVERICK-HCM and VALOR-HCM, the extended follow-up durations offered by these investigations are intended to provide increased clarity regarding the degree of adverse medication effects in the long run [26], [27]. However, a decrease in the ejection fraction is a potential adverse consequence of Mavacamten because it is a myosin inhibitor and as such has a detrimental ionotropic impact. However, only 6.0 % and 3.6 % of patients in the EXPLORER-HCM and VALOR-HCM studies, respectively, were found to have an ejection fraction of less than 50 %, and all of these cases improved after taking the medicine off-label for a while [11], [14]. The Risk Evaluation and Mitigation Strategy (REMS) program further explores the ramifications of this adverse effect with regular echocardiograms scheduled within 21–28 days of the first dose for the patient population. Echocardiograms are further scheduled after 4 weeks and in the maintenance phase. A fall of ejection fraction of more than 50 % warrants temporary cessation of the drug until ejection fraction increases again [28]. With the recent approval of a novel drug mavacamten for the treatment of HCM, it is important to establish the efficacy and safety profile of the drug before establishing it as a first line treatment for HCM patients. Firstly, further long term trials are required to investigate the long term adverse effects of the drug such as atrial fibrillation and heart failure due to its negative inotropic effect on the cardiac muscle. The implication of mavacamten in causing long term adverse effects in HCM patients is still under study. Mavacamten's DISCOVER-HCM registry (NCT05489705) is anticipated to enroll about 1500 patients with oHCM and evaluate the drug's efficacy and safety in the real world in the United States. Its role in heart failure with preserved ejection fraction will be investigated in the EMBARK-HFpEF trial (NCT04766892) and in non-oHCM heart failure in the ODYSSEY-HCM trial (NCT05582395). Furthermore, out of the four completed RCTs, only the MAVERICK-HCM trial employed a patient population of non-obstructive HCM patients; therefore, subgroup analysis was not sufficient to differentiate the significance of the results for obstructive and non-obstructive HCM patients [11].

Another important aspect regarding the use of mavacamten and HCM is the familial link of HCM with the MYH7 mutation. Mavacamten was first approved for patients having the MYH7 mutation, though this genetic factor was not included in the inclusion criteria of the trials. Further investigations should be carried out to explore this correlation [29]. All of the current literature has been focused on an adult population of HCM patients; investigations targeted towards prophylactic treatment of children predisposed to HCM should be employed. The future open label and trial studies on Mavacamten will give us more information about its safety and efficacy in pregnancy [30], [31], [32], efficacy in HNCM (ODYSSEY-HCM), effect on coronary flow reserve in HOCM, and single dose with and without activated charcoal with sorbitol [33], [34].

## 2 Strengths and limitations

This *meta*-analysis is the only robust *meta*-analysis analyzing four latest RCTs published on mavacamten’s efficacy. Sub-studies and extension studies of significant RCTs were used in earlier *meta*-analyses' outcome analysis, which adversely affected the accuracy of their findings. The establishment of the safety profile of mavacamten will be significantly impacted by our investigation, which is the only review to analyze serious side events individually.

There are various limitations in this *meta*-analysis that should be taken into account. First off, the included studies had a variety of designs, lengths of time, and patient groups, which could have caused the results to be heterogeneous. Ho et al. and Desai et al. investigated the effect of mavacamten with a 16 week follow up which significantly differed from the other three included studies’ 32 week followup period which could have contributed to the heterogeneity amongst the included trials [14], [15]. Second, the available trials did not thoroughly assess mavacamten's long-term effects on clinical outcomes like mortality and the risk of sudden cardiac death. To close this knowledge gap, more extensive follow-up periods will be used in future investigations. Our study did not stratify the data for obstructive and non-obstructive HCM separately due to inadequate data which may lead to generalizability of the results.

In conclusion, Mavacamten clinically improves symptoms in HCM patients by at least 1 grade in the NYHA class and KCCQ CSS score improvement with no significant side effect profile.

## CRediT authorship contribution statement

**Irfan Ullah:** Data curation, Conceptualization. **Syeda Tayyaba Rehan:** Writing – original draft, Formal analysis. **Zayeema Khan:** Methodology, Investigation. **Syed Hasan Shuja:** Resources, Methodology, Data curation. **Muhammad Hamza Shuja:** Formal analysis, Data curation. **Muhammad Irfan:** Writing – review & editing, Validation. **Karthik Gonuguntla:** Writing – original draft, Resources. **M Chadi Alraies:** Writing – review & editing, Project administration. **Pratik Aggarwal:** Visualization, Data curation. **Sameer Raina:** Writing – review & editing, Validation, Software. **Yasar Sattar:** Writing – review & editing, Visualization, Supervision. **Muhammad Sohaib Asghar:** Writing – review & editing, Formal analysis.

## Declaration of competing interest

The authors declare that they have no known competing financial interests or personal relationships that could have appeared to influence the work reported in this paper.
